# Follicular Lymphoma Presenting with Leptomeningeal Disease

**DOI:** 10.1155/2014/767621

**Published:** 2014-12-02

**Authors:** Rubens Costa, Ricardo Costa, Renata Costa

**Affiliations:** ^1^Cedar Valley Cancer Center, 3530 West 4th Street, Waterloo, IA 50701, USA; ^2^Department of Oncology, Real Hospital Portugues, 52010 Recife, PE, Brazil; ^3^American University of the Caribbean, Coral Gables, FL 33134, USA

## Abstract

Follicular lymphoma is generally an indolent B cell lymphoproliferative disorder of transformed follicular center B cells. Central nervous system metastasis is a very rare complication portending a very poor prognosis. We report a rare case of follicular lymphoma presenting with leptomeningeal involvement achieving a complete remission after initial therapy.

## 1. Introduction

Central nervous system (CNS) involvement by aggressive non-Hodgkin's lymphoma (NHL) is a well-described complication portending a very poor prognosis [1, 2]. Reports of CNS metastases by indolent NHL are scarce [2–4]. We report a rare case of follicular lymphoma presenting with diffuse lymphadenopathy, osseous lesions, and simultaneous leptomeningeal involvement.

## 2. Case

The patient was a 52-year-old healthy male who presented to an ophthalmologist and neurologist with left side ptosis, headache, and photophobia. His initial working diagnosis was myasthenia gravis. A magnetic resonance imaging (MRI) and magnetic resonance angiogram (MRA) of the head showed no abnormality. Antibodies for acetylcholine receptor antibody were positive. Pyridostigmine was started with short lived improvement. The patient also established care with a new primary care physician with the complaints above, arthralgias, a 40-pound weight loss, and profound asthenia. A complete blood count and metabolic profile was within the normal range except for mild hyponatremia. Lactate dehydrogenase (LDH) was at least 5 times above the upper limit of normal. Erythrocyte sedimentation rate (ESR) and C reactive protein (CRP) were elevated.

Twelve days later the patient presented to the emergency room with intractable headache and bony pain. On physical examination, his vital signs were normal. Visual acuity was impaired. Pupils were poorly reactive with anisocoria. The left pupil was fixed and did not react to light. His ocular motility was impaired bilaterally. Bilateral ptosis was noted. Indirect ophthalmoscopy showed bilateral papilledema with hemorrhage. The rest of the neurologic examination was normal except for the third cranial nerve palsy. Axillary as well as inguinal lymphadenopathy was present on physical examination.

**Figure 1:**
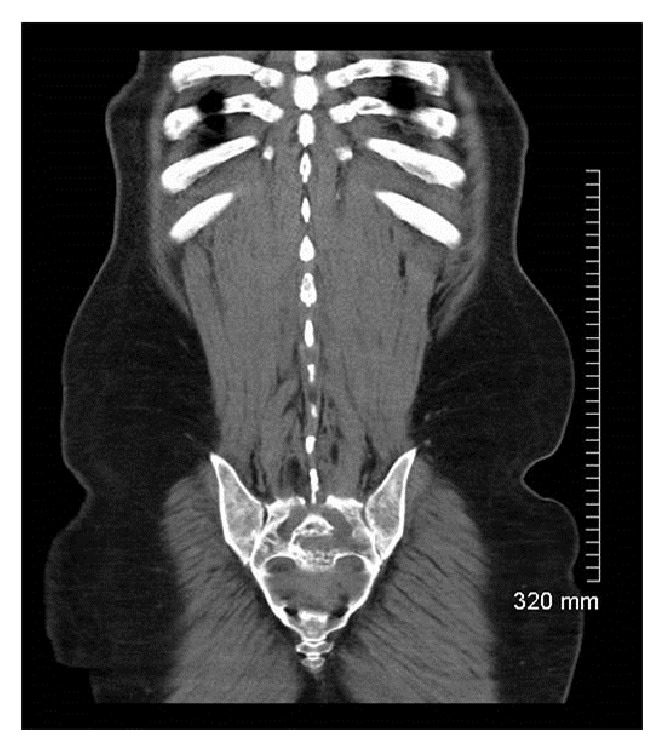
Computerized tomography showing a presacral mass.

**Figure 2:**
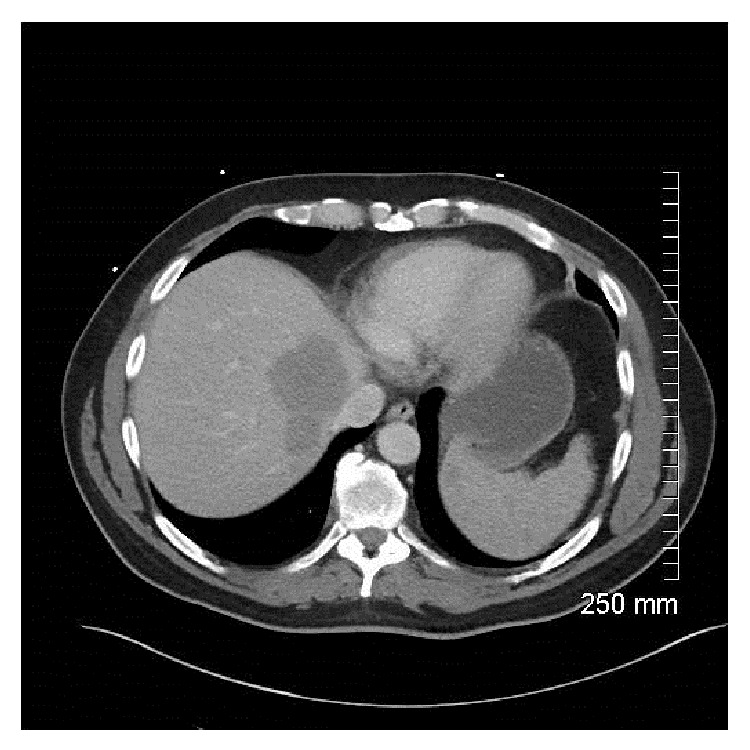
Computerized tomography showing a liver mass.

Computerized tomography (CT) of the chest, abdomen, and pelvis revealed right axillary, left inguinal, and retroperitoneal lymphadenopathy. Presacral and liver masses were noted (Figures 1 and 2). There were multiple osteolytic bony lesions. MRI of the brain, cervical, and thoracic spines revealed diffuse osseous metastatic lesions. Subtle patchy areas of enhancement were noted along the cord. Linear enhancement was noted along the third and fifth cranial nerves.

**Figure 3:**
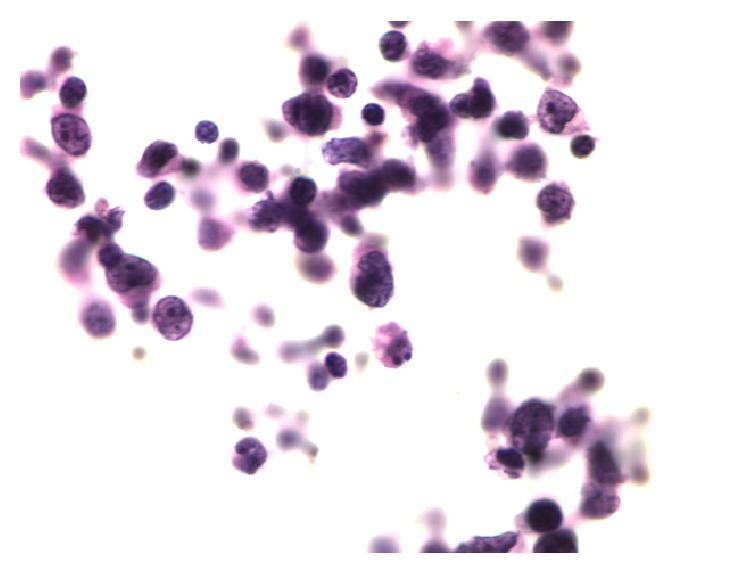
Spinal fluid showing atypical lymphoid cells with cytologic features consistent with follicular lymphoma.

**Figure 4:**
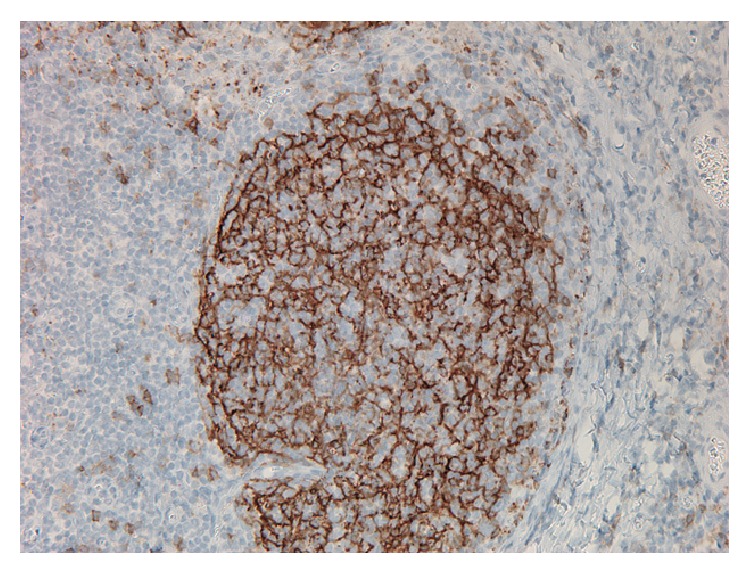
Lymphoid cells strongly expressing CD23.

A right inguinal lymph node biopsy showed variable degree of nodular proliferation of atypical lymphoid cell proliferation composed predominantly of centrocytes with admixed scattered centroblasts (approximately ten per high power field). The neoplastic nodules were positive for CD20, CD10, bcl-6, and bcl-2. Ki-67 highlighted approximately 10–20% of neoplastic lymphoid cells. The cells stained positive for CD23 confirming a follicular origin (Figure 3). Flow cytometry analysis revealed a CD10 positive, CD19, CD20 positive, kappa immunoglobulin light chain restricted B cell population. The results were consistent with follicular lymphoma grade 2 (Figure 4). A bone marrow biopsy was not performed.

**Figure 5:**
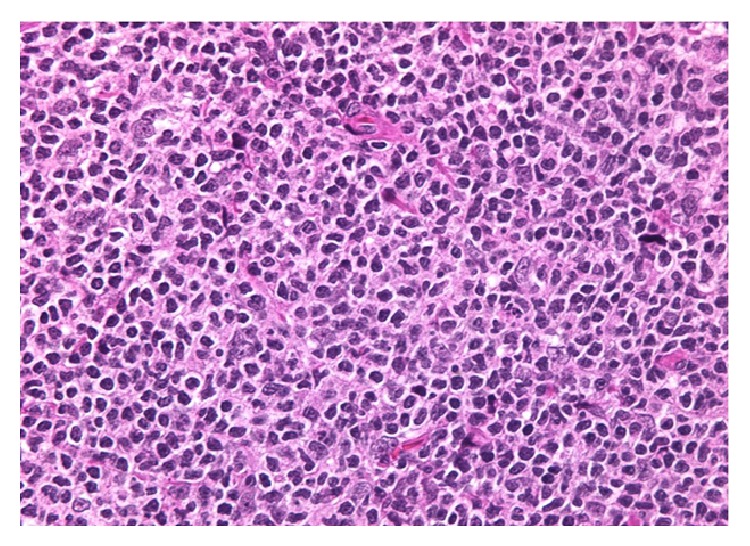
Nodular proliferation of atypical lymphoid cells composed predominantly of centrocytes with admixed scattered centroblasts.

Cerebrospinal fluid (CSF) was hazy. Cell count showed 253 cells/mm [3]. Glucose was 17 mg/dL (range, 45–75 mg/dL). Protein level was 265 mg/dL (range, 15–45 mg/dL). Cytology analysis revealed atypical lymphoid cells with cytologic features consistent with the previously diagnosed follicular lymphoma on lymph node biopsy (Figure 5).

Therapy was started with the R-MTX/Ara-C/Hyper-CVAD regimen every 21 days. His treatment was as follows: intravenous (iv) methotrexate 200 mg/m^2^ over 2 hours followed by 800 mg/m^2^ continuous infusion over 22 hours on day 1 and cytarabine 3 g/m^2^ (iv) over 2 hours every 12 hours for 4 doses on days 2 and 3. This regimen was alternated with cyclophosphamide 300 mg/m^2^ (iv) over 2 hours every 12 hours for 6 doses, vincristine 2 mg on days 4 and 11, adriamycin 50 mg/m^2^ (iv) over 24 hours on day 4, and dexamethasone 40 mg daily on days 1–4 and days 11–14. Rituximab 375 mg/m^2^ was administered with all six cycles of treatment every 21 days. His CSF cleared after one instillation of methotrexate of a total of 8. His symptoms and neurologic finding completely resolved. A positron emission tomography after completion of therapy confirmed a complete remission. He refused evaluation for high dose chemotherapy followed by autologous stem cell rescue as consolidation therapy. He remained free of symptoms 12 months after original diagnosis.

## 3. Discussion

Follicular lymphoma is generally an indolent B cell lymphoproliferative disorder of transformed follicular center B cells. It is the most common subtype of indolent NHL and comprises about 22% of all cases. Follicular lymphoma is characterized by diffuse lymphadenopathy, marrow involvement, splenomegaly, and less commonly extranodal sites. It is not considered curable with current standard therapies except for, in highly selected patients, allogeneic stem cell transplantation [5].

CNS involvement by NHL occurs in approximately 8% of the cases. Predictive factors are aggressive histology, such as Burkitt's and lymphoblastic lymphomas; paranasal sinuses and testicular involvement; and adverse prognostic features such as increased LDH, bone marrow involvement, and multiple extranodal sites involvement. CNS involvement by indolent lymphoma is typically described comprising a very small percentage of large lymphoma series and case reports. This complication occurs in 3% mainly after histological transformation to high-grade lymphoma. Chemotherapy, radiation, surgery, or a combination of these modalities has been used as part of its treatment [3, 6–12].

Our case is a rare presentation of follicular, symptomatic, systemic, and leptomeningeal disease as evidenced by clinic, laboratory, radiology, pathology, and cytology data. His presentation with neurologic symptoms and bulky symptoms required prompt treatment after diagnosis while being hospitalized. A PET/CT scan could not be performed prior to start of therapy. A bone marrow evaluation was deferred because of proven stage 4 disease. We chose an anthracycline containing regimen alternating with methotrexate/cytarabine in an attempt to properly address his systemic and CNS disease.

In our opinion, the most relevant aspect of this case is the fact that the cells found in the spinal fluid were morphologically similar to the cells on his lymph node biopsy compatible with follicular lymphoma. Disease transformation was a strong diagnostic consideration due to clinical symptoms, disease bulk and laboratory data. Because of his presentation, there was not enough time for another biopsy to prove a different histology prior to treatment.

In conclusion, this case represents one of the rare reports of a probable transformed follicular lymphoma with its indolent component causing leptomeningeal disease and neurologic symptoms.
